# Mental health of Brazilian students during the COVID-19 pandemic: the role of gratitude, optimism, and hope in reducing anxiety

**DOI:** 10.47626/2237-6089-2022-0496

**Published:** 2024-04-23

**Authors:** Joice Franciele Friedrich Almansa, Tatiane Trivilin, Claudio Simon Hutz, Rosa Maria Martins de Almeida, Ana Claudia Souza Vazquez, Clarissa Pinto Pizarro de Freitas

**Affiliations:** 1 Programa de Pós-Graduação em Psicologia Universidade Federal do Rio Grande do Sul Porto Alegre RS Brazil Programa de Pós-Graduação em Psicologia, Universidade Federal do Rio Grande do Sul (UFRGS), Porto Alegre, RS, Brazil.; 2 Núcleo de Estudos de Psicologia Positiva Organizacional e do Trabalho UFCSPA Porto Alegre RS Brazil Núcleo de Estudos de Psicologia Positiva Organizacional e do Trabalho, Universidade Federal de Ciências da Saúde de Porto Alegre (UFCSPA), Porto Alegre, RS, Brazil.; 3 Programa de Pós-Graduação em Psicologia e Saúde UFCSPA Porto Alegre RS Brazil Programa de Pós-Graduação em Psicologia e Saúde, UFCSPA, Porto Alegre, RS, Brazil.; 4 Pontifícia Universidade Católica do Rio de Janeiro Rio de Janeiro RJ Brazil Curso de Psicologia, Pontifícia Universidade Católica do Rio de Janeiro (PUC-Rio), Rio de Janeiro, RJ, Brazil.

**Keywords:** Anxiety, college students, pandemic, positive psychology, protective factors

## Abstract

**Objective:**

To investigate the role of optimism, hope, and gratitude as psychosocial factors for healthy development, especially with regard to anxiety in college students in the context of coronavirus disease 2019 (COVID-19).

**Methods:**

This is a quantitative, descriptive cross-sectional study. A sociodemographic questionnaire, the Brazilian Gratitude Scale (Escala Brasileira de Gratidão [B-GRAT]), and the Brazilian versions of an anxiety subscale, the Revised Life Orientation Test (LOT-R), Hope Index Scale, and BIG-FIVE were administered. Data were analyzed using the Mann-Whitney *U* test, the Kruskal-Wallis test, Spearman correlations, and hierarchical linear regression.

**Results:**

A total of 297 students were assessed. The relationship between gratitude and anxiety became positive in the hierarchical linear analysis, contradicting the initial negative association between these variables according to the Spearman coefficients. This contradiction may be a result of the suppression effect. When gratitude was added to the model, these three variables together accounted for 38% of the variance in anxiety. This indicates that optimism, hope, and gratitude together are significant predictors, although optimism alone accounts for the greater part of the variance in decreased anxiety.

**Conclusion:**

The data confirm that family and religiosity are protective factors against mental illness, specifically non-adaptive anxiety. Furthermore, developing optimism as a protective factor makes it possible to experience less anxiety while hope has the potential to provide the individual with multiple pathways to healthy development. This study highlights that gratitude plays a dual role in these relationships as it has the potential to be associated with anxious feelings with likely negative outcomes while at the same time it can drive positive psychosocial factors of optimism and hope, decreasing anxiety.

## Introduction

Higher education students’ mental health is being studied in many different countries. There has been a high prevalence of mental disorders,^1^ psychological stress (26.2%),^2,3^ depression (17.3%), suicidal ideation, and self-injurious behavior (15.3%).^4^ In the European context, the World Health Organization (WHO)^5^ reported an increase in mental illnesses in 11% of the population, reaching 10 to 20% of young adults.

Research has shown that the coronavirus disease 2019 (COVID-19) pandemic has caused psychological illnesses, such as depression and anxiety,^6,7^ generating fear, or coronaphobia.^8^ Current studies demonstrate considerably increased anxiety in students during the COVID-19 pandemic. A relationship was detected between anxiety and cognitive functioning before and during COVID-19.^9,10^ Studies in Brazil showed an increase in anxiety (39.7%) and prevalence of moderate-severe symptoms, including research with a sample of students.^11-13^

Anxiety is characterized by fear or extreme tension that exceeds healthy levels and brings harm and illness. Since university is a new environment that requires emotional and social adaptation, feelings, sensations, and insecurities can emerge, which have the potential to compromise students’ learning and their relationship with professors, colleagues, and the university itself. Difficulties arise with reconciling study schedules, internships, exams, and personal, professional, and family life.^14,15^

In a study with 257 college students, it was found that students’ positive perception of their life also reflects positively on their physical, psychological, social relationship, and environment domains, increasing the frequency of positive affect and reducing adverse effects.^16^ In this regard, the positive psychology movement aims to (re)build positive qualities and foster and nurture the best in subjects, focusing on healthy factors, virtues, and personal strengths as protective and preventative agents of mental illness.^17,18^ Thus, hope, gratitude, and optimism are constructs that approach this purpose and are potential predictors of mental health in college students.^19-21^

Hope is defined as a positive emotional state present in a triad formed by objective (the search for something), route (the possibility of a path to reach the goal), and agency (the motivation to trace the route and achieve the objective).^22^ Magaletta and Oliver^23^ found that agency is a better predictor of subjective well-being. For Merolla and Peterson,^24^ hope was negatively associated with the amount of daily interpersonal conflict, positively related to constructive conflict management when a battle occurred, and negatively related to everyday challenges in maintaining relationships.

Moreover, some studies show that higher levels of hope were associated with greater well-being and perceived emotional control, as well as with lower levels of perceived COVID-19 anxiety and stress.^25^ Also, hope could decrease students’ focus and insecurity during COVID-19^20^ and help them to reduce procrastination in the educational process by creating new routes when faced with adversity,^19^ yielding higher intellectual satisfaction and engagement.^26^ Shellstrom,^27^ reflects on the lessons from remote learning, including the importance of adaptability during the pandemic and appreciation of little things crucial for better academic well-being, especially in critical moments. Algoe^28^ emphasizes that gratitude is also aimed at the individual’s awareness of their life experiences, realizing their positive side.

Based on evidence from the Brazilian population, Vazquez et al.^29^ defined gratitude as a positive mental state of gratitude for life experiences, even if adverse or risky, linked to affective memories that enhance subjective well-being. The researchers found that gratitude is positively associated with hope, explaining 38% of variance, and indicated that gratitude is a protective and preventive factor against anxiety and depression.

Moreover, in an experiment with students during the pandemic,^30^ Puente-Diáz and Cavazos-Arroyoin showed that bringing to mind memories of special moments has a positive influence on gratitude and mediates the positive relationship between recalling a special event and optimism. These findings showed that these constructs might help people see a brighter future under the prevailing difficult conditions of prolonged lockdowns.

Evidence has shown that optimism promotes positive mental strategies. Optimistic individuals employ self-confidence and more significant effort and persistence to achieve their goals using thought control and logical analysis of adversity or perceived risk.^31^ Furthermore, researchers identified that coronavirus stress was negatively associated with college students’ sense of hope and optimism. These mitigated the adverse impacts of tension on mental health during the pandemic. It was concluded that being hopeful and optimistic are the potential resources that explain how coronavirus stress is related to subjective well-being.^32^

In the present study, we consider that hope has the potential to provide students with multiple routes of positive development, even in the face of anxiety and difficulties.^24^ Thus, hope has a negative association with anxiety levels (hypothesis 1). Also, gratitude can promote reflection and elaboration of negative feelings and increase students’ well-being by experiencing positive feelings, even in adverse situations or when the routes followed do not suit their initial expectations.^29^ Therefore, gratitude is negatively related to anxiety, acting as a psychosocial protective factor (hypothesis 2).

Moreover, optimism can provide a positive vision of the future, even in adversity, and optimistic students will be successful in their actions, achieving the goals they aspire to.^32^ Thus, optimism is negatively related to anxiety levels (hypothesis 3). In our view, the complexity of this process will enable the student to achieve the growth necessary for their personal and professional development in a healthy way. Therefore, optimism will act as the main protective factor in decreasing anxiety in comparison with hope and gratitude (hypothesis 4).

Given the above, the main contribution sought in this article is to investigate the roles of optimism, hope, and gratitude as psychosocial factors to strengthen, boost, and protect healthy development, especially regarding anxiety in college students in the context of COVID-19. Such evidence from the present study could contribute to well-being interventions in university students.

## Methods

This is cross-sectional, quantitative and descriptive research. Data were collected using SurveyMonkey software to send emails containing invitations to participate in the study to educational institutions and companies. Data collection took place throughout Brazil from April 11 to June 23, 2020. The study is part of a larger project that included other studies to investigate the link between positive psychology and mental health.

### Participants

The sample comprised 297 individuals from the student population, aged between 18 and 77 years (mean = 29.43, standard deviation = 9.87), mostly women (78.79%), college students (77.40%), and single (76.77%).

### Instruments

#### Brazilian version of the Revised Life Orientation Test (LOT-R)^33^

The LOT-R contains 10 items with a Likert response scale (ranging from strongly disagree = 1 to agree strongly = 5) and has an adequate reliability index (α = 0.79). In the present study, reliability (α = 0.97) and LOT-R indexes were satisfactory (χ^2^ = 13.9, degrees of freedom [df] = 3, p < 0.001, root mean square error of approximation [RMSE] = 0.06 [90% confidence interval {90%CI} = 0.03-0.09], goodness-of-fit index [GFI] = 0.99, comparative fit index [CFI] = 0.99, normed fit index [NFI] = 0.99, and incremental fit index [IFI] = 0.99).

#### The anxiety scale comprised the anxiety-specific items from the Emotional Adjustment/Neuroticism Factor Scale^34^

This anxiety subscale has 17 Items with a Likert response scale ranging from 1 to 5 (1 = strongly disagree to 5 = strongly agree) and with an α coefficient of 0.89. In the sample assessed for this research, internal consistency (α = 0.92) and observed fit indices were satisfactory (χ^2^ = 495.8, df = 106, p < 0.001, RMSEA = 0.06 [90%CI 0.06-0.07], GFI = 0.94, CFI = 0.96, NFI = 0.95, and IFI = 0.96).

#### The Brazilian version of the Hope Index Scale^35^

This scale consists of 12 items distributed in two subscales: Agency Thoughts (items 2, 9, 10, and 12) and Path Thoughts (items 1, 4, 6, 8), measured on a five-point Likert scale, (1 = totally false to 5 = totally true, with α = 0.92). In the sample assessed for this research, the internal consistency α was 0.86. The results of the confirmatory factor analysis indicated that the model has adequate statistical fit (χ^2^ = 53.7, df = 16, p < 0.001, RMSEA = 0.05 [90%CI 0.03-0.06], GFI = 0.98, CFI = 0.98, NFI = 0.98, and IFI = 0.98).

#### Brazilian Gratitude Scale (B-GRAT)^29^

The B-GRAT consists of seven items, five items measuring gratitude towards external sources, i.e. people, things, and God, plus two items assessing the dispositional state. Responses are scored on a five-point Likert scale, where 1 represents false and 5 true, with a Cronbach’s alpha of 0.84. In this study, the scale presented adequate fit indices (χ^2^ = 59.9, df = 12, p < 0.001, RMSEA = 0.06 [90%CI 0.05-0.08], GFI = 0.98; CFI = 0.98; NFI = 0.98; IFI = 0.98) and internal consistency (α = 0.85).

## Data analysis procedures

The Statistical Package for the Social Sciences (SPSS) version 25 was used for data analysis. Analysis of missing data was conducted and participants who did not complete all four scales were excluded. Outliers were later analyzed using the procedure suggested by Hair et al.^36^ to calculate the Mahalanobis distance, which measures the deviations of values from the means of predictor variables. Cases were excluded that had d^2^/df values significant to p < 0.001. On this basis, the cutoff point is 22.46.

Cook’s distance was calculated. This measure considers the effect of a single case on the model as a whole by calculating 4/(NK-1), where k is the number of predictors in the model and n is the number of participants. The cutoff point adopted was 0.004. The leverage distance was used to verify the mean value of influence by calculating (2K + 2)/N, with the cutoff value set at 0.014. After calculating the three distances, values greater than or equal to each cutoff point were coded as 1 and values below the cutoff were coded 0 and the results for all three distances were summed, defining ≥ 2 as the overall cutoff.

The Shapiro-Wilk test (SW) and Kolmogorov-Smirnov test (K-S) were applied. These tests assume the hypothesis of data normality (hypothesis 0), returning a p-value > 0.05 if the results fit normal parameters, while significant values (p < 0.05) indicate deviation from normality. Multicollinearity was analyzed by checking each variable’s tolerance, which measures the proportion of its variance that is not explained by the remaining independent variables. If the tolerance of a variable is < 0.1, then there is multicollinearity between the variables, which must be excluded from the model. The variance inflation factor (VIF) was also used, which is the inverse of tolerance. If the VIF is smaller than 10, there is no collinearity and the closer it approaches to 0, the smaller the collinearity.^37,38^

Since data were not normally distributed, Mann-Whitney *U* tests were used for non-parametric quantitative variables with dichotomous categories and Kruskal-Wallis tests were used for variables with more than two groups. The significance level adopted was p < 0.05. Cohen’s *d* was calculated for all significant group comparisons to indicate effect size.^39^

A biserial point correlation was used to investigate associations between sociodemographic features (marital status, sex, and religious practice) and anxiety, hope, gratitude, and optimism. Spearman correlation coefficients were calculated to identify associations between psychological variables. Hierarchical linear regression analysis was employed to determine whether adding variables significantly improves a model’s ability to predict the criterion variable and investigate the moderating effect of variables.

## Ethical aspects

The Universidade Federal do Rio Grande do Sul research ethics committee approved the research project (CAAE:80264617.3.0000.5334). All participants signed an informed consent form prior to data collection.

## Results

Thirty-two participants were excluded because of missing data, Mahalanobis distance, Cook’s distance, and/or leverage distance. The K-S test was highly significant for the values of all four constructs, indicating that the distributions deviate from normality. The anxiety D (297) = 0.56, p < 0.05, optimism D (297) = 0.97 p < 0.001, hope D (297) = 0.06, p < 0.05, and gratitude D (297) = 0.12, p < 0.001 results were all significantly non-normal. Deviation from normality requires use of non-parametric tests. Transformation of variables could have been employed, but criticism of artificiality could arise. It was therefore decided not to transform the variables and employ multivariate techniques, notwithstanding recognition that generalization of results is compromised. VIF values are below 10 for the current model, and tolerances are all above 0.20. Therefore, we can safely conclude that there is no collinearity within the data.

Participants were categorized according to sociodemographic variables, gender (female and male), and marital status, (‘no spouse’ for single or widowed, and ‘has spouse’ for married or in a relationship). Religious practice and having children were also treated as dichotomous variables. Finally, university education was classified as undergraduate, postgraduate certificate, master’s, or doctorate (Table 1).

**Table 1:** Sociodemographic data

Mann-Whitney *U*		Anxiety		Optimism		Hope		Gratitude	
Sex	n	M (SD)	Z (*d*)	M (SD)	Z (*d*)	M (SD)	Z (*d*)	M (SD)	Z (*d*)
Female	234	3.25 (0.83)	-4.2* (0.24)	3.58 (0.95)	0.005 (0.00 )	3.78 (0.69)	-0.61 (0.04)	4.15 (0.75)	-3.91* (0.23 )
Male	63	2.75 (0.80)		3.56 (0.98)		3.73 (0.73)		3.65 (0.92)	
Marital status									
No spouse	228	3.22 (0.83)	-3.90^†^ (0.23 )	3.48 (0.94)	-3.63* (0.21 )	3.72 (0.70)	-2.18^†^ (0.13)	4.00 (0.83)	-1.64 (0.10)
Has spouse	69	2.88 (0.83)		3.93 (0.90)		3.93 (0.67)		4.19 (0.75)	
Religious practice									
Yes	130	3.02 (0.83)	-2.09^†^ (0.12 )	3.81 (0.87)	-3.48* (0.20)	3.94 (0.63)	-3.37^†^ (0.20)	4.41 (0.68)	-7.22* (0.42)
No	164	3.24 (0.85)		3.41 (0.99)		3.64 (0.72)		3.78 (0.79)	
Children									
Yes	51	2.79 (0.91)	-3.05^†^ (0.18)	3.91 (0.88)	-2.80 (0.16)	3.93 (0.79)	-1.77^†^ (0.10)	4.30 (0.81)	-2.85^†^ (0.17)
No	246	3.21 (0.82)		3.51 (0.95)		3.74 (0.67)		3.99 (0.81)	
Kruskal-Wallis									
University level									
Undergraduate	230	3.19 (0.84)	9.097 (0.53)^†^	3.54 (0.95)	8.235 (0.48)^†^	3.73 (0.69)	4.778 (0.28)	4.03 (0.81)	4.156 (0.24)
Postgraduate certificate	21	2.61 (0.70)		4.09^†^ (0.85)		4.05 (0.59)		4.28 (0.91)	
Master’s	32	3.13^†^ (0.85)		3.67^†^ (0.87)		3.73 (0.72)		4.11 (0.77)	
Doctorate	14	3.05 (0.93)		3.37 (1.16)		3.85 (0.91)		3.84 (0.86)	

*d* = *d* de Cohen; M = mean; SD = standard deviation; Z = score z.

* Correlation is significant at the 0.01 level.

^†^ Correlation is significant at the 0.05 level.

The comparison between sociodemographic variables, anxiety, optimism, hope, and gratitude demonstrated that females had higher levels of anxiety and gratitude than males. Regarding marital status, those who had no spouse had a higher level of anxiety, lower optimism, and lower hope than those who did have a spouse. Moreover, participants who endorsed no religious practice had higher levels of anxiety and lower levels of optimism, hope, and gratitude than those who did not. On the other hand, students who did observe religious practice had lower anxiety levels and increased optimism, hope, and gratitude. Finally, participants who had children had lower levels of anxiety and higher levels of optimism and gratitude than those who did not have children (Table 1).

In relation to academic level, undergraduates had higher levels of anxiety, followed by students studying for master’s degrees, doctorates, and postgraduate certificates. On the other hand, postgraduate certificate students had higher levels of optimism, followed by master’s, undergraduates, and, finally, doctorate students (Table 1).

Spearman coefficients were calculated to identify correlations between the study variables. First, the correlation analysis showed that sex was negatively related to anxiety and gratitude. Also, age was negatively correlated with anxiety and there were positive correlations between age and optimism, hope, and gratitude, which means that younger people had higher anxiety levels and less optimism, hope, and gratitude than older adults (Table 2).

**Table 2:** Associations between sociodemographic variables with anxiety, optimism, hope, and gratitude

	Anxiety	Optimism	Hope	Gratitude
Sex	-0.248*^†^	0.000	-0.035	-0.227*
Age	-0.248*	0.225*	0.219*	0.198*
University level	-0.102^‡^	0.076	0.096^‡^	0.035
Marital status	-0.219*^†^	0.207*	0.131^‡^	0.148*
Religious practice	0.122*^†^	-0.204*	-0.196*	-0.422*
Children	0.178*^†^	-0.163*	-0.103^‡^	-0.165*
Anxiety	1	-0.594*	-0.375*	-.214*
Optimism		1	0.542*	0.502*
Hope			1	0.526*
Gratitude				1

Children = 1: yes, 2: no; Marital status = 1: no spouse; 2: has spouse; Religious practice = 1: yes, 2: no; Sex = 1: female, 2: male; University level = 1: undergraduate, 2: postgraduate certificate, 3: master, 4: doctorate.

* Correlation is significant at the 0.01 level (1-tailed).

^†^ Point biserial correlations; all others are Spearman correlations.

^‡^ Correlation is significant at the 0.05 level (1-tailed).

University level was negatively related to anxiety and positively significant with regard to hope, which means that people with lower educational levels could be less anxious and more hopeful. Marital status was negatively related to anxiety and positively related to optimism, hope, and gratitude. Finally, religious practice and having children were both negatively related to anxiety and positively related to optimism, hope, and gratitude. In other words, people who are religious, people who are in romantic relationships, and those who have children are less anxious than those that are not (Table 2).

Moreover, it can be observed that anxiety was negatively related to optimism, hope, and gratitude. Optimism showed the strongest negative association with anxiety (-0.594, p < 0.001), followed by hope (-0.375, p < 0.001), and then gratitude (-0.214, p < 0.001). Hope and optimism had the strongest positive relationship with each other (0.542, p < 0.001) and gratitude was strongly related to hope and optimism (0.526, 0.502, p < 0.001, respectively). These findings are shown in Table 2.

Hierarchical regression was performed to investigate the contribution of optimism, hope, and gratitude to explaining anxiety levels. However, this analysis entails certain assumptions. The Durbin-Watson value calculated for this study is 2.081. Based on this value, there is no problem with multicollinearity or autocorrelation among these variables.

It was found that all predictor variables added to the model significantly predicted less anxiety and, according to the hierarchical regression analysis results, just two of the models were significant. According to this, the variables most associated with decreasing anxiety were optimism, hope, and gratitude, in that order. According to the hierarchical regression analysis results from the first model, optimism alone predicted 34% of anxiety decrease. Adding the variable hope to the model, these two variables together accounted for 35% of the variance in anxiety, although this model was not significant. Furthermore, when gratitude was added, these three variables together accounted for 38% of the variance in anxiety. This indicates that together optimism, hope, and gratitude are significant predictors, but that optimism alone accounts for the greater part of the variance in decreased anxiety (Table 3).

**Table 3:** Regression model for predicting anxiety in predictive variables (dependent variable: anxiety)

Model	Anxiety	*B*	R	R^2^	R^2^ Adj.	R change	Sig	Durbin-Watson
1	Optimism	-0.521	0.587*	0.345	0.343	0.345	0.0001	
2	Optimism	-0.469	0.593^†^	0.352	0.348	0.007	0.074	
	Hope	-0.124						
3	Optimism	-0.526	0.617^‡^	0.380	0.374	0.028	0.0001	2.081
	Hope	-0.218						
	Gratitude	0.216						

* Predictors = (constant), Optimism.

^†^ Predictors = (constant), Optimism, Gratitude.

^‡^ Predictors = (constant), Optimism, Gratitude, Hope.

## Discussion

Sociodemographic data indicated that people in a romantic relationship have lower anxiety scores and higher levels of optimism and hope. Moreover, it was observed that people who engage in religious practice had lower anxiety levels and higher levels of optimism, hope, and gratitude. Having children was also associated with lower anxiety scores and increased levels of optimism and gratitude. The data confirm that family and religiosity are protective factors against mental illness, specifically in this research into non-adaptive anxiety.^40^

The highest levels of anxiety were observed in undergraduate and master’s students. Studies carried out in university settings have corroborated this evidence. In a meta-analysis, Chang et al.^41^ noted a high prevalence of anxiety among college students during the COVID-19 pandemic. Moreover, Ibrahim et al.^42^ demonstrated that the high rates of anxiety and depression in students, especially in the first years at university, are even higher than those found in the general population. On the other hand, undergraduate and master’s students demonstrated low levels of optimism. This shows the theoretical and practical relevance of thinking about strategies for coping with obstacles and strengthening positive effects, since they are necessary actions for well-being and emotional balance.^41-43^

Moreover, optimism and hope were negatively associated with anxiety confirming hypotheses 1 and 3. Also, optimism acted as the main protective factor in decreasing anxiety in comparison with hope and gratitude, as predicted by the fourth hypothesis. A negative correlation between anxiety and optimism was demonstrated in findings reported by Hutz and Nunes^34^ and by Bastianello et al.^33^ Furthermore, a negative association with hope was found in studies by Gadosey et al.^44^ and Long and Gallager.^45^ In the correlation data, it is possible to observe that optimism, hope, and gratitude are positively related to each other, confirming the findings of Witvliet et al.,^46^ Kardas et al.,^47^ Vazquez et al.,^29^ and Biber et al.^48^

However, it was notable that the relationship between gratitude and anxiety becomes positive in the hierarchical linear analysis, contradicting these variables’ negative association indicated by the Spearman coefficients. These contradictory results in the analysis may be a result of the suppression effect,^49^ showing that the Spearman correlation analysis of the indirect effects of gratitude, optimism, hope, and anxiety hinders the direct effect of gratitude on anxiety. Importantly, the direct effect is more informative than the indirect effect because it considers the relationship between variables, considering the different influences on this relationship.^49^

It is important to consider that these data were collected during the first 2 months of the pandemic in Brazil, since this initial period saw a notable increase in anxiety levels.^7,8,50^ Based on our evidence, we observed that in COVID-19 times, students experienced fear of loss of things they were grateful for, such as health, family members, and study. That is, the more gratitude they had for things and people, the greater their fear of losing them. Thus, gratitude seems to exert a toxic effect in times of adversity and possible loss, being positively associated with anxiety.

Our findings corroborate those of Davis et al.,^51^ Jans-Beken et al.,^52^ and Cregg and Cheavens,^53^ who found no negative correlations between gratitude and anxiety. Other studies have found that gratitude increases well-being and decreases anxiety. In this sense, it can be seen that gratitude acts with a positive effect on hope and optimism, significantly increasing the explanatory power of the model. Gratitude, hope, and optimism are constructs primarily studied in the positive psychology field, which shows evidence that their development as predictors of mental health favors individual strengthening and emotional balance, thus increasing the capacity to respond more satisfactorily to the challenges and adversities faced in life.^29,48,54^

The scientific literature presents robust findings showing that even in difficult times development of optimism as a protective factor makes it possible to experience less anxiety,^33,55^ while hope has the potential to provide individuals with multiple routes of healthy development, even when facing anxiety and other difficulties.^23,56^ In our study, we highlight evidence that gratitude plays a dual role in these relationships, since it has the potential to be associated with anxiogenic feelings with likely negative outcomes while it can also boost positive psychosocial factors of optimism and hope, decreasing anxiety.

## Conclusions

The pandemic in Brazil has been linked with evidence of many psychological illnesses, such as anxiety and depression. The aim of this research was therefore to evaluate the roles of gratitude, optimism, and hope, as psychosocial protective factors for reducing anxiety, and thus in improving the mental health of college students during the COVID-19 pandemic. The hypotheses that hope and optimism would correlate negatively with anxiety and that optimism would play a major role compared to the other variables were both proven. However, in relation to gratitude, a positive association with anxiety was observed in the hierarchical analysis, making it impossible to confirm hypothesis 2 that gratitude would decrease anxiety. Nevertheless, an indirect action was attributed, via hope and optimism, decreasing anxiety. In pandemic or high adversity situations, development of optimism associated with hope and gratitude will have more protective effects on people’s well-being and also be more effective in reducing anxiety.

This paper aims to contribute current data to enable greater understanding of these constructs, realizing that they also have limitations and adverse effects when they do not confirm the importance of psychosocial protective factors such as hope, gratitude, and optimism, as well as family and religious practice, to encourage students to improve their mental health and well-being, in line with what is proposed by positive psychology regarding optimal functioning to reduce anxiety in order to maintain adequate adaptive levels in adverse contexts. These data can also be used to support practitioners, teachers, and universities thinking about better strategies to improve students’ mental health and to create effective interventions based on findings from this pandemic context.

Some limitations of the present research include the predominance of undergraduate females in the sample. Also, data were collected during a pandemic situation and a majority of the sample was from Brazil’s southern region. However, it is important to study how these variables interact in males and in master’s and doctorate students. Moreover, research in non-pandemic situations and longitudinal study designs would help understand how these variables interact in other contexts.

It is suggested that further studies be carried out to analyze the effects of optimism, hope, and gratitude on factors such as anxiety and depression, as well as positive factors related to proactivity. Furthermore, replicating this research in a non-pandemic situation would be of great value for better understanding of the constructs. It is also suggested that interventions based on the findings of this study could be implemented to verify the results in practical experiences.
